# Time to Go Back to the Original Name

**DOI:** 10.3389/fmicb.2017.01800

**Published:** 2017-09-22

**Authors:** Robert C. Gallo, Luc Willems, Yutaka Tagaya

**Affiliations:** ^1^Division of Basic Science, Institute of Human Virology, University of Maryland School of Medicine Baltimore, MD, United States; ^2^HTLV-1 Task Force, Global Virus Network Baltimore, MD, United States; ^3^Research Director, National Fund for Scientific Research at University of Liège Liège, Belgium

**Keywords:** HTLV-1, T-cell leukemia, myelopathy, oncogenic viruses, retrovirus

Almost 40 years ago, the first pathogenic human retrovirus was discovered and shown to cause a T-cell leukemia in humans (Poiesz et al., 1980a, 1981). Thus, a new paradigm was established showing that a virus can directly cause cancer in humans. At that time, the only other suspected case was the connection between the Epstein-Barr Virus (EBV) and Burkitt's Lymphoma and later nasopharyngeal carcinoma. Naturally, and in keeping with the names used for animal retroviruses associated with leukemia, we (the Gallo group) named this new virus as human T-cell leukemia virus (HTLV), which was formally accepted by the scientific community. As of today, our list of human oncogenic viruses has expanded to include hepatitis B/C virus (HBV, HCV), papillomaviruses (HPV), Herpes virus-8 (HHV-8), and Merkel cell polyomavirus (MCV). In parallel, the group of Delta-retroviruses that HTLV belongs to has expanded to accommodate additional members over the years, now consisting of HTLV-1~4, and STLV-1~4. In the meantime, the name of the original virus was changed to human T-lymphotropic virus-1, because of the addition of the viral causative agent of AIDS as HTLV-III to the same group (Coffin, 2015). Thus, one of us (RCG) agreed on this name change with several other retrovirologists at a Cold-Spring Harbor meeting in 1983. In retrospect, the name change has made it ambiguous as to which virus should enter this group, and since the AIDS virus was formally named HIV and separated from the HTLV group, making the need for the name of HTLV-1 as human T-“lymphotropic” virus no longer particularly useful or meaningful. Finally, we note here the greater oncogenicity of HTLV-1 compared to other viruses, or even other known carcinogens which is a point in keeping a name that describes their effect (discussed in an accompanying article). Therefore, we propose to restore the original name “leukemia virus.”

## Lymphotropic vs. leukemia; a historical background

The original names for HTLV-1 came from two groups in association with an unusual type of human T-cell leukemia (adult T-cell leukemia) (Poiesz et al., 1980a,b; Hinuma et al., 1981). At first two names existed; “Human T-cell leukemia virus (-I)” and “Adult T-cell leukemia virus.” A study in 1982 confirmed the identical nature of these two viruses (Popovic et al., 1982) and a meeting was held to unify “names.” US and Japanese researchers agreed on acknowledging the US priority for the discovery of the virus and the Japanese priority in defining the very particular type of leukemia (Yodoi et al., 1974; Uchiyama et al., 1977). Hence, the virus adopted the name “Human T-cell leukemia” and the disease “adult T-cell leukemia.” Around this time, a genetically related (70% structural similarity to HTLV-1) virus was identified in association with a CD8 T-cell variant of a hairy cell leukemia (Kalyanaraman et al., 1982) and this virus was added to the group as HTLV-II. A turn of event followed shortly that another virus originally thought to be closely related to the HTLV group was discovered in connection with AIDS (Popovic et al., 1984), a virus now known as HIV-1. HIV-1 was originally regarded as another member of HTLVs and added to the group as HTLV-III (Gallo et al., 1983). As mentioned above, these events led to the change of the naming from “Leukemia” to “Lymphotropic” (Coffin, 2015). The following years have seen explosive research on HIV-1 and soon HTLV-III was shown to be more substantially different from HTLV-I/II. Eventually a new name, HIV-1, was given to HTLV-III and HIV-1 was taxonomically separated from HTLVs. It has become also clear that HTLV-1 does not only infect T cells (Koyanagi et al., 1993). Thus, the first name change has lost its ground and perhaps the nomenclature should have been reversed to the original at much earlier point.

## HTLV-1 as a potent carcinogen

HTLVs cause not only leukemia, but also HAM/TSP, a progressive and incurable myelopathy (Nagai and Osame, 2003). Disease manifestations caused by HTLV-1 also include inflammatory arthropathy and uveitis. Because of this diverse clinical impact, there have been arguments even in the HTLV-1 community if the “leukemia” name is the most appropriate. We note several points; (1) Precedent throughout animal retroviruses and notably these animal retroviruses also can cause non-neoplastic disorders as well as leukemias/lymphomas, (2) Precedent as the first formal name for HTLV, (3) the fact that leukemia is the most frequent severe outcome of HTLV-1 infection, and (4) the fact that viruses commonly cause more than one disease as noted above. There are some suggestions that the name “leukemia” might be overly intimidating, given that over 80% of the HTLV-1-infected individuals remain disease-free for their lifetime. We are of course aware of the challenges imposed on infected people and clinicians who are in charge of caring the infected individuals. However, we note that HTLV-1 is one, if not the most, potent oncogenic virus and therefore the name “leukemia virus” has scientific significance that outweighs other concerns. We have recently seen reports involving the Japanese Ministry of Health and Labor which declared to target HTLV-1 as one of the major focuses in 2010 that they revised the prevalence of HAM/TSP in Japan down to 0.3% whereas that of ATL remains 4–5% as proposed before, which suggests that ATL indeed represents more than 80% of the disease manifestations caused by HTLV-1 in Japan. Moreover, this argument flies in the face of names for most other disease-causing agents (e.g., influenza, polio, hepatitis, human immunodeficiency virus, Kaposi's sarcoma herpes virus, tuberculosis, etc.). Finally, in an age when patients have easy access to information, the point seems moot. It is of note that unlike animal oncoviruses, not necessarily all human known oncoviruses include the direct mention to cancer. Merkel cell carcinoma virus and Kaposi-sarcoma herpes virus (HHV-8) have direct words in their names. Hepatitis B and C viruses were identified as viruses causing hepatitis and later linked to carcinogenesis. Epstein-Barr virus and Papilloma viruses (HPV) were isolated from cancer cell/tissues, but took a long time to establish the link to cancer. The HTLV-1 represents a unique exception. The peculiar geographical distribution of adult-T cell leukemia (Yodoi et al., 1974; Uchiyama et al., 1977) promoted researchers to suspect that a virus might be the cause of it. Moreover, there are many precedent connecting retroviruses and leukemogenesis in animals (hence many animal retroviruses cause leukemia carry the name “leukemic” in their names). The discovery of HTLV-1 was thus a consequence of “hunting for a leukemia-causing virus.” Moreover, the causative link of the discovered virus to ATL was quickly established within a few years after the discovery. This makes a pivotal success of modern virology and therefore, we feel compelled to commemorate the relevance of these events by renaming HTLV-1 as the original “leukemia virus.”

The oncogenic association of HTLV-1 to ATL is unquestionable. Once diagnosed as an acute phase or lymphomatous phase of ATL, the patient has less than 12 months of life left, indicating the aggressive nature of the oncogenic property of HTLV-1 as well as the lack of effective treatment to this fatal disease caused by HTLV-1, though there are promising treatments emerging. In comparison, the oncogenic capacity were not as convincing for other members of the HTLV family. However, the original identification of HTLV-2, which is not highly oncogenic, came from a CD8 T-cell variant of Hairy Leukemia cells. STLV-associated leukemia among monkeys has been reported (Miura et al., 2013). There is no concrete evidence to strongly link HTLV-2, -3, and -4 with particular human malignant diseases. Indeed some of the earlier epidemiology studies failed to show strong correlation between HTLV-2 and the T-cell type hairy cell leukemia (Quesada et al., 1986; Rosenblatt et al., 1988). HTLV-2 encodes Tax-2 gene which is structurally highly similar to the oncogenic Tax-1 gene of HTLV-1. However, recent studies suggest that another gene called HBZ (HTLV-1 bZIP factor) which is encoded by the minus-strand of the HTLV-1 can cause an ATL-like CD4 leukemia in mice (Satou et al., 2011). Interestingly, HTLV-2 encodes a similar anti-sense gene (Anti-sense viral protein of HTLV-2, APH-2), but the structure and function of APH-2 seems very different and this may account for the differences of oncogenic properties of HTLV-1 and -2 (Panfil et al., 2016). However, an alternative to the above explanation might be connected to the tropism of these two viruses. HTLV-1 is clearly CD4 T-cell tropic whereas HTLV-2 is mainly detected in CD8 T cells (Ijichi et al., 1992; Xie and Green, 2005; Jones et al., 2006). HTLV-1 does not transform CD8 T cells though it infects CD8 T cells as well at the early phase of infection. This may reflect the fundamental differences of intracellular pathways required for cellular transformation in CD4 and CD8 T cells, rather than the differences between HTLV-1 and -2. Consistently, transgenic experiments over-expressing so-called T-cell growth cytokines such as IL-15 and IL-7 results in the development of T-cell leukemia in these mice but that is only limited to CD8 (and NK) lineages (Rich et al., 1993; Fehniger et al., 2001; Sato et al., 2011). It is of note that CD4 T cells in these mice show robust DNA synthesis (Marks-Konczalik et al., 2000) but never become leukemic. Conversely, we only see leukemia of CD4 T cells by HTLV-1. It is likely that that mechanism by which HTLVs transform host cells works efficiently in CD4 T cells, but not in CD8 T cells. If this were true, HTLV-2 can be leukemic, but not very efficient in CD8 T cells. This view contrasts with the former argument which regards HTLV-2 “non-oncogenic because of the lack of molecular machinery.” In fact, recent studies demonstrate that Tax-2 of HTLV-2 transforms T cells as efficient as does Tax-1 *in vitro* (Ren and Cheng, 2013). Another recent publication demonstrates that HTLV-2 facilitates clonal expansion of CD4 or CD8 T cells in infected individuals. The same study showed that the resultant clone frequency resembles that in established ATL than the frequency in asymptomatic carriers of HTLV-1 (Melamed et al., 2014). Needless to say, clonal expansion is often associated with cancers. These pieces of argument may not provide a strong justification to call HTLV-2 an oncogenic virus, but surely suggest that the issue is still pending. We also need more data about HTLV-3 and 4 to determine their pathogenicity. In conclusion, we only limit the renaming proposal to one member, the HTLV-1, leaving other members for a final decision in the future.

In an accompanying manuscript (Tagaya and Gallo, 2017), we also discuss an intriguing finding from a survey of recent literature that HTLV-1 is the most oncogenic virus known. We therefore propose that the name of this virus should clearly represent the concept by including the word “leukemia” in it. We recently conducted a survey among the HTLV-1 experts who belong to the HTLV-1 task-force of the Global Virus Network (http://gvn.org). GVN represents a coalition of virologists on a global scale, and its HTLV-1 task-force includes major HTLV-1 researchers from all-over the world (member roster shown in Willems et al., 2017). A vote was made on the following four choices; (1) to keep the lymphotropic virus as its name (2) to re-adopt the leukemia virus name, (3) to change it to Primate T-lymphotropic virus-1, (4) to change it to Primate T-cell leukemia virus-1, and the result was 5 for (1), and 16 for (2) (none for 3 and 4). The vast majority therefore supported readopting the original name “Human T-cell leukemia virus-1.” In addition, a similar poll conducted at the 18th International Conference on Human Retrovirology (Tokyo, 2017) showed 78 vs. 26 in support of the “leukemia” name. We consider these votes compelling because the former (the GVN vote) reflects the majority opinion of the HTLV experts in the research community and the latter (Tokyo, 2017) represents the community opinion of many HTLV-1 researchers from all over the world.

We thus propose the renaming of HTLV-1 as “human T-cell leukemia virus-1” to commemorate an illustrious event in the history of modern virology.

## Author contributions

All three authors conceptualized and discussed the idea and accordingly LW and RG conducted the first poll among GVN's HTLV-1 task force members. Then LW organized a poll at the 18th International Retrovirology meeting in Tokyo (2017). The three wrote the manuscript together.

### Conflict of interest statement

The authors declare that the research was conducted in the absence of any commercial or financial relationships that could be construed as a potential conflict of interest.
